# Lack of Evidence of Chikungunya Virus Infection among Blood Donors during the Chikungunya Outbreak in Lazio Region, Italy, 2017

**DOI:** 10.3390/v14030619

**Published:** 2022-03-16

**Authors:** Giulietta Venturi, Massimo Fabiani, Antonello Amendola, Giulia Marsili, Eleonora Benedetti, Cristiano Fiorentini, Claudia Fortuna, Simonetta Pupella, Patrizio Pezzotti, Stefania Vaglio, Giulio Pisani, Vincenzo De Angelis, Flavia Riccardo, Ilaria Pati

**Affiliations:** 1 Department of Infectious Diseases, Italian National Institute of Health, 00161 Rome, Italy; giulietta.venturi@iss.it (G.V.); massimo.fabiani@iss.it (M.F.); antonello.amendola@iss.it (A.A.); giulia.marsili@iss.it (G.M.); eleonora.benedetti@iss.it (E.B.); cristiano.fiorentini@iss.it (C.F.); claudia.fortuna@iss.it (C.F.); patrizio.pezzotti@iss.it (P.P.); flavia.riccardo@iss.it (F.R.); 2 National Blood Centre, Italian National Institute of Health, 00161 Rome, Italy; simonetta.pupella@iss.it (S.P.); stefania.vaglio@guest.iss.it (S.V.); vincenzo.deangelis@iss.it (V.D.A.); 3 Department of Molecular Medicine, Sapienza University, 00185 Rome, Italy; 4 National Centre for the Control and Evaluation of Medicines, Biologicals and Biotechnologicals Unit, Italian National Institute of Health, 00161 Rome, Italy; giulio.pisani@iss.it

**Keywords:** chikungunya virus, Italian outbreak, blood donation, viraemia, Lazio Region

## Abstract

Background: The latest European Chikungunya virus (CHIKV) outbreak occurred in Italy in 2017, in the municipalities of Anzio and Rome (Lazio Region), with a secondary outbreak in the Calabrian Region. Most CHIKV infections are symptomatic but about 15% of people who acquire the infection may be asymptomatic. A retrospective study was conducted with the aim of assessing the prevalence of recent/ongoing CHIKV infections on the blood donor population in the Lazio Region, during the 2017 outbreak (including in the period before it was detected). Methods: The study was conducted on 4595 plasma samples from donors who donated in 14 different Blood Establishments in the Lazio Region, in the period June–November 2017. A total of 389 of these samples were collected in provinces not affected by the outbreak and were used as negative controls. All samples were tested for IgM detection by the use of an ELISA test, and positive samples were tested for confirmation through the use of a PRNT. Molecular tests were performed on sera that were found to be IgM-positive or borderline. Results: A total of 41 (0.89%) blood donors tested positive for IgM. None of these positive IgM ELISA results was confirmed either by PRNT or by molecular tests. Conclusions: Our study has shown no evidence of recent/ongoing CHIKV infection in blood donors of the affected area.

## 1. Introduction

Chikungunya fever (CHIKF) is a vector-borne disease, caused by an RNA virus belonging to the *Togaviridae* family, genus *Alphavirus*, transmitted to humans through the bite of infected female mosquitoes of the genus *Aedes*. The Chikungunya virus (CHIKV) disease is characterized by acute fever and severe joint pain [1,2]. Serious neurological, ocular, cardiac and gastrointestinal complications have occasionally been reported [3]. Most CHIKV infections are symptomatic but approximately 15% of infections are asymptomatic [4].

CHIKV infection is a public health problem in many African and Asian countries [5]. Over the past decade, it has also become a threat to public health in temperate areas colonized by *Aedes* mosquitoes, such as Europe and the Americas [6]. Climate change and travel to endemic areas, have contributed to the spread of CHIKV [7].

The first CHIKV outbreak in Europe occurred in 2007 in Italy, in the Emilia Romagna Region, with 337 notified human cases, 217 of which were laboratory-confirmed. Local transmission was supported by the vector *Aedes albopictus* and the case index was a man from an epidemic area of India (Kerala). The outbreak affected the provinces of Ravenna and Forlì-Cesena, with other small clusters detected in other areas of the Region [5,8,9].

Subsequent CHIKV outbreaks in Europe affected France in the years 2010, 2014 and 2017 [5,10,11]. In 2017, ten years after the first outbreak, the latest European CHIKV outbreak occurred in Italy in the Lazio Region, in the municipalities of Anzio and Rome, with a secondary outbreak in the Calabrian village of Guardavalle Marina [12,13]. In total, 499 probable cases were notified, 270 of which were laboratory-confirmed.

The first three patients with a history of high fever (>38 °C), severe joint pain and an itching skin rash, were diagnosed with CHIKV infection on 6 and 7 September 2017 [14].

The epidemiological analysis of the outbreak in the Lazio Region suggested the occurrence of three main foci of local transmission. The major cluster involved 317 cases with an epidemiological link with the Anzio municipality. The other two clusters occurred in the municipalities of Rome (80 cases) and Latina (8 cases) [15]. Based on epidemiological data and on a transmission model using mosquito abundance and biting rates, the first imported case was estimated to be in the period between May and June 2017 [16]. The earliest symptom onsets were retrospectively detected starting from late June, suggesting a considerable delay in the notification of first cases. The latest symptoms onset of the last case occurred in early November 2017 [15]. To date, the transmission of CHIKV infection by blood transfusion has not been documented [17], although Nucleic Acid Test (NAT) screening in the Caribbean in 2014 showed that it is possible to detect pre-symptomatic viraemic blood donors in a high prevalence context in the absence of limitations [18,19]. During the 2007 outbreak in Emilia Romagna Region, the epidemic in the Lazio Region had significant repercussions on the Italian blood system [9,20].

In 2017, as in 2007, preventive measures were immediately introduced to reduce the risk of enrolling asymptomatic positive CHIKV donors, and to ensure the blood supply in the affected areas, despite the absence of an authorized screening test for blood donations. These measures included: the local interruption of blood collection from donors living in the affected areas (district 2 of the Rome municipality and the municipality of Anzio), a 5-day quarantine of red blood cells collected from donors with a history of travel in the other districts of the municipality of Rome, reinforcement of donor clinical assessment and post-donation information for donors who had travelled in the affected areas or who resided in the Lazio region.

In addition, a national level 28-day deferral of donors who reported travelling/living in the municipalities of Anzio and Rome was applied [20]. Plasma for fractionation was collected without limitations, while platelets and plasma for clinical use were transfused after pathogen inactivation [20].

According to a common internationally approved approach, the European statistical classification of the affected areas (regional—NUT2, and provinces—NUT3) was routinely used to identify the geographical extension of the outbreak. During the 2017 CHIKV outbreak, the interruption of blood collection was limited to the affected district areas (1,300,000 resident population) due to the unsustainable impact of applying those measures to the entire municipality of Rome (2,783,809 resident population).

Laboratory diagnosis of the CHIKV infection is mainly based on molecular assays to detect the CHIKV genome from blood, and serological tests that include ELISAs for the detection of IgM and IgG anti-CHIKV antibodies in serum. Due to the possible cross-reactivity among different arboviruses, the more specific Plaque Reduction Neutralization test (PRNT) is used to confirm the ELISA test-positive results. Indeed, according to the ECDC case definition, the confirmation of a CHIKV case requires a positive molecular or PRNT result when analyzing a single serum sample [21].

CHIKV genomic RNA is already detectable in the pre-symptomatic phase (one or more days before the onset of symptoms), and becomes undetectable within 7 days [17], while IgM antibodies are usually detectable in serum 5–7 days after onset of symptoms and persist for about 4 months. From 1 to 2 days after IgM seroconversion, the IgG antibodies are detected [22].

Considering the risk of future CHIKV outbreaks in Italy, and their potential impact on the blood system, a retrospective study was conducted with the aim of assessing the prevalence of ongoing/recent CHIKV infections in the blood donor population of the Provinces of the Lazio Region affected by the 2017 outbreak, and, consequently, an assessment of the effectiveness of the preventive measures introduced that did not include Nucleic Acid Testing (NAT) screening.

## 2. Materials and Methods

We conducted a cross-sectional study in order to assess the prevalence of recent/ongoing CHIKV infections among 4595 plasma samples from donors who had donated in 14 different Blood Establishments (BEs) in the Lazio region during the period of viral circulation (June–November 2017).

These BEs were serving four of the five regional provinces (i.e., Rome, Latina, Viterbo, and Rieti). Of those, 11 BEs were serving areas in the provinces of Rome and Latina that were affected by the CHIKV outbreak in 2017. The catchment areas of the three remaining BEs were the two provinces of Viterbo and Rieti, where CHIKF cases were not detected. Out of 4595 plasma samples, 389 belonged to donations collected in the two unaffected provinces. These samples were used as negative controls.

### 2.1. Laboratory Testing

Blood donations were tested for the presence of CHIKV-specific IgM antibodies by an ELISA test. Positive IgM samples were also tested by real-time PCR and by a PRNT for confirmation.

#### 2.1.1. Serological Assays Anti-CHIKV IgM ELISA Test

IgM antibodies against CHIKV were detected using the commercial Euroimmun anti-CHIKV IgM ELISA system (Euroimmun Medical Laboratory Diagnostics AG, Lübeck, Germany). The test was performed according to manufacturer’s instructions. Absorbance was measured at 450 nm using an ELISA iMark™ Microplate Absorbance Reader (Bio-Rad Laboratories, Hercules, CA, USA). Sample optical density (O.D.) readings were compared with reference cut-off OD readings to determine results. Reading values ≥ 1.1 were considered presumptive for the presence of IgM antibodies, values ≥ 0.8 < 1.1 were considered borderline, and values < 0.8 were considered negative.

#### 2.1.2. Plaque Reduction Neutralization Test (PRNT)

A PRNT was performed, as previously described, by Fortuna et al. [23]. Briefly, the assay was performed in six-well tissue culture plates with subconfluent VERO cell monolayers and a CHIKV strain isolated from a patient, during the 2007 Italian outbreak [6]. Serum samples were diluted 1:10 in serum-free maintenance medium; equal volumes (100 μL) of CHIKV dilution containing approximately 80 Plaque Forming Units (PFU), and serum dilutions, were mixed, and incubated overnight at +4 °C. After 1h incubation at +37 °C and 5% CO_2_, the inoculate was aspirated and the wells were overlayed with a mixture of one part 2% Gum Tragacanth and one part of supplemented medium (2× MEM, 2.5% inactivated FCS and 2% 1 M HEPES). The plates were incubated at +37 °C and 5% CO_2_ for 2 days, and then were stained with 1.5% crystal violet. A titration of CHIK viruses with three dilutions in duplicate (the working dilution, 1:2 and 1:8 dilutions) was performed in each assay, and used as a control for the assay. Neutralizing antibody titers were calculated as the reciprocal of the serum dilution that gave an 80% reduction in the number of plaques (PRNT80), as compared to the virus control. PRNT80 ≥ 10 were considered positive.

#### 2.1.3. RNA Extraction and Real-Time RT-PCR

A molecular test was performed on IgM-positive or borderline sera. RNA was extracted from 200 μL of serum sample using QIAmp Viral RNA Mini Kit (Qiagen Inc., Hilden, Germany), according to the manufacturer’s instructions. To detect the presence of viral genomic RNA, real-time RT-PCR was performed using 7 μL of RNA, with primers CHIKS, CHIKAs and probe ChikP, and SensiFAST Probe No-ROX One-Step Kit (Meridian Bioscience, Memphis, TN, USA) according to the manufacturer’s protocol. Amplification was performed by CFX96 Touch™ Real-Time PCR Detection System (Bio-Rad) [6,23].

### 2.2. Statistical Analysis

We estimated the prevalence of ELISA-IgM-positive and PCR/PRNT-confirmed sera together with exact binomial 95% confidence intervals (CI). Prevalence was estimated both overall and stratifying by calendar period (i.e., June–July, August–September, and October–November 2020) and geographical area (i.e., areas affected vs. areas not affected by CHIKV cases outbreak in summer 2017).

The analysis was performed using Stata/SE version 16.0 (StataCorp LLC, College Station, TX, USA).

## 3. Results

The demographic characteristics of the whole population of blood donors who attended in the year 2017 in the 11 selected BEs, serving areas affected by the CHIKF outbreak, were very similar to those of the population of blood donors from the three selected BEs serving areas that were not affected (Table 1). Overall, about 30% of the blood donors were female, and about 56% were aged 36–55 years, with only a small proportion of elderly donors aged 65 years or more (0.3%).

Overall, 0.89% (41/4595) of samples had a positive ELISA IgM test result and 1.6% (74/4595) had a borderline result. Table 2 shows the prevalence of CHIKV IgM positivity by calendar period and geographical area, among the 4595 blood donors included in the study. No significant differences in seroprevalence were observed according to the calendar period of blood donation and the provenience of the plasma.

None of the 115 positive and borderline sera was positive upon PCR testing. A PRNT was performed on all 41 CHIKV IgM-positive samples and in a subset of 48/74 borderline serum samples, in order to assess the presence of CHIKV-specific neutralizing antibodies, with no positive results. Positive results obtained by using the Euroimmun anti-CHIKV IgM ELISA test were thus considered as non-specific.

Overall, CHIKV prevalence among the 4595 blood donors was 0% (97.5% CI: 0–0.08%).

## 4. Discussion

Although most CHIKV infections are symptomatic, about 15% of people who acquire the infection may be asymptomatic. Further, viraemia can precede symptom onset [4]. Studies conducted in 2014, during the Puerto Rico epidemic, reported that 0.54% of asymptomatic viraemic donors had a viral load between 2.9 × 10^5^ and 9.1 × 10^7^ copies/mL [24]. A case-control study, conducted during the 2009 CHIKV epidemic in Thailand, did not report significant differences between the highest viremia values detected in symptomatic positive subjects, compared to RNA positive asymptomatic subjects [4]. This evidence suggests that asymptomatic or presymptomatic viraemic individuals could potentially transmit CHIKV through transfusion. The identification of CHIKV viraemic plasma samples from pre-symptomatic donors through NAT screening in Martinique in 2014 seems to support this hypothesis, as does the risk-estimate of viraemic donation calculated in the studies conducted during the epidemics in Thailand (risk between 38 and 52 out of 100,000 donations) and in Réunion (average risk equal to 132 out of 100,000 donations) [25,26].

Notwithstanding, to date, no case of transfusion-transmitted CHIKV infection has been documented [17].

The 2017 CHIKV outbreak in the Lazio Region, with its secondary outbreak 600 km to the South, focused attention on the risk of re-emergence and endemization of this infection in Italy, especially considering that the competent vector *Aedes albopictus* is largely established in the country [13,14,15].

The first cases were diagnosed at the beginning of September 2017. However, the earliest symptom onsets were retrospectively dated back to early June, revealing a 2–3-month delay in the detection of the outbreak.

In the absence of a validated molecular test for blood donor screening, measures to prevent transmission through transfusion were based on the deferral of donors travelling/living in the areas in which CHIKV infections had been detected, and on the suspension of blood component donations in affected areas [20].

Outbreaks, as observed in Lazio, Calabria and Emilia Romagna, are possible in the future in the same, or in other, Regions in Italy. This could also be the case in other countries where a competent vector is largely established. By retrospectively assessing evidence of CHIKV contamination among blood donations collected in the 2017 most-affected Italian Region during the period of known local circulation, we investigated whether contaminations reflected the 2017 CHIKV spread in order to better assess the risk of CHIKV transmission through a blood transfusion during the outbreak.

In doing this, we assessed the effectiveness of the introduced control measures in limiting the risk of CHIKV transmission through blood transfusions.

We chose to preliminarily screen blood donations for the presence of CHIKV-specific-IgM antibodies, which are associated with recent/ongoing infections, using an ELISA test, the most convenient in terms of costs and simplicity of execution. IgM antibodies can be detected in blood for several months after infection, unlike the viral genome that is only detectable for a few days. IgM-positive samples were subsequently tested using a molecular test and the PRNT for confirmation. With this approach, we aimed to detect blood donors who had contracted a CHIKV infection a few days to a few months before donation.

Overall, our results showed that only 0.89% of all samples tested positive for IgM. A borderline ELISA IgM result was obtained for 1.6% of the tested samples. None of the samples testing positive or borderline were later confirmed by PCR to be contaminated by CHIKV. As expected, the subset of borderline samples tested by the PRNT was also negative. The observed 0.89% of non-specific positive ELISA IgM results are in line with data from the literature, and with the 0.8% reactive rate reported in the package insert of the Euroimmun anti-CHIKV IgM ELISA system for a panel of 498 blood donors [27,28,29,30].

These findings excluded the presence of ongoing recent infections in our study, supporting transfusion safety, and also as a consequence of the effectiveness of the adopted control measures in limiting transfusion risks during the 2017 epidemic. Several factors may have favoured the success of the adopted measures: the high proportion of clearly symptomatic cases associated with human CHIKV infection, together with the short incubation period and the typical cluster distribution of cases due to transmission dynamics. All these factors would make the persistent lack of detection of at least one human case in an area (e.g., a province) where viral circulation is ongoing unlikely once the outbreak was known [13]. Therefore, activating measures in areas in which at least one human case had been observed, likely was effective in limiting donations from the population most likely to be infected. Interestingly, no samples were found to be positive in the early months of transmission (June–August) before the outbreak was detected and control measures were implemented. Possibly, an initially limited circulation among younger populations played a role.

Currently, authorized NAT is available for blood donation screening. Its implementation in case of a possible new outbreak, would allow for the detection of asymptomatic infected blood donors. This approach could reduce transfusion risk without interrupting blood collection in the affected areas, deferring donors or quarantining red blood cell components.

A limitation of our study is that by choosing to preliminarily screen blood donations for the presence of CHIKV-specific IgM antibodies, we could have missed pre-symptomatic/asymptomatic patients in the short time frame (usually a few days) between exposure to the virus and the development of an IgM antibody response. However, as we could not find any confirmed viraemic samples across all those tested, we assume it is unlikely that this limitation could substantially invalidate our findings.

## 5. Conclusions

Despite the significant spread of the CHIKV 2017 outbreak in the Lazio Region, our study has shown no evidence of recent/ongoing CHIKV infections in the studied blood donor population. These results also support transfusion safety, as a consequence of the effectiveness of the measures implemented across the Italian blood system.

## Figures and Tables

**Table 1:** Demographic characteristics of the populations of blood donors from the Blood Establishments in the areas included in the study in the year 2017.

	Donors from Areas Affected by CHIKF in 2017		Donors from Areas Not Affected by CHIKF in 2017		Overall	
	No.	%	No.	%	No.	%
**Age bracket**						
18–25 years	11,445	13.9	2237	14.3	13,682	14.0
26–35 years	13,387	16.3	2908	18.6	16,295	16.7
36–45 years	21,765	26.5	4138	26.5	25,903	26.5
46–55 years	25,038	30.5	4224	27.1	29,262	29.9
56–65 years	10,327	12.6	2031	13.0	12,358	12.6
>65 years	190	0.2	57	0.4	247	0.3
**Sex**						
Female	25,275	30.8	4281	27.5	29,556	30.2
Male	56,877	69.2	11,314	72.5	68,191	69.8
**Total**	82,152	100.0	15,595	100.0	97,747	100.0

**Table 2:** Prevalence of Chikungunya virus among 4595 blood donors in the Lazio region (Italy, June–November 2017).

		Positive IgM-ELISA			Borderline IgM-ELISA			Positive or Borderline IgM-ELISA			Positive PCR-Confirmed		
Calendar Period/Geographical Area	No. Tested	No.	%	(95% CI) *	No.	%	(95% CI) *	No.	%	(95% CI) *	No.	%	(97.5% CI)
**June–July 2017**													
Affected areas	1689	14	0.83	(0.45–1.39)	26	1.54	(1.01–2.25)	40	2.37	(1.70–3.21)	0	0.00	(0.00–0.22)
Unaffected areas	156	3	1.92	(0.40–5.52)	7	4.49	(1.82–9.03)	10	6.41	(3.12–11.5)	0	0.00	(0.00–2.34)
**August–September 2017**													
Affected areas	1338	7	0.52	(0.21–1.07)	16	1.20	(0.69–1.93)	23	1.72	(1.09–2.57)	0	0.00	(0.00–0.28)
Unaffected areas	49	0	0.00	(0.00–7.25)	0	0.00	(0.00–7.25)	0	0.00	(0.00–7.25)	0	0.00	(0.00–7.25)
**October–November 2017**													
Affected areas	1179	14	1.19	(0.65–1.98)	24	2.04	(1.31–3.01)	38	3.22	(2.29–4.40)	0	0.00	(0.00–0.31)
Unaffected areas	184	3	1.63	(0.34–4.69)	1	0.54	(0.01–2.99)	4	2.17	(0.60–5.47)	0	0.00	(0.00–1.98)
**Overall period (June–November 2017)**													
Affected areas	4206	35	0.83	(0.58–1.16)	66	1.57	(1.22–1.99)	101	2.40	(1.96–2.91)	0	0.00	(0.00–0.09)
Unaffected areas	389	6	1.54	(0.57–3.33)	8	2.06	(0.89–4.01)	14	3.60	(1.98–5.96)	0	0.00	(0.00–0.94)
**Total**	4595	41	0.89	(0.64–1.21)	74	1.61	(1.27–2.02)	115	2.50	(2.07–3.00)	0	0.00	(0.00–0.08)

CI, confidence interval. * One-sided 97.5% CI where prevalence is 0%.

## Data Availability

The data are available at the Department of Infectious Diseases, Italian National Institute of Health, Rome, Italy.
